# Late T‐wave inversion following resolution of non‐ischemic acute pulmonary edema

**DOI:** 10.1002/ccr3.1899

**Published:** 2018-11-12

**Authors:** Konstantinos Tampakis, Nikolaos Makris, Christos Kontogiannis, Michael Spartalis, Evangelos Repasos, Eleftherios Spartalis, Hector Anninos, Ioannis Paraskevaidis

**Affiliations:** ^1^ Department of Clinical Therapeutics, Medical School "Alexandra" Hospital, National and Kapodistrian University of Athens Athens Greece; ^2^ Division of Cardiology Onassis Cardiac Surgery Center Athens Greece; ^3^ Laboratory of Experimental Surgery and Surgical Research, Medical School University of Athens Athens Greece

**Keywords:** acute pulmonary edema, hypertension, myocardial ischemia, T‐wave inversion

## Abstract

Electrocardiographic (ECG) changes occurring several hours after the onset of acute cardiogenic pulmonary edema have been seldom described. The proposed explanatory mechanisms are various and not fairly established. In the absence of significant coronary artery disease, these ECG abnormalities could be attributed to mechanisms implicated in coronary microcirculatory dysfunction.

## 1 CASE PRESENTATION

A 65‐year‐old male presented to our emergency department with extreme breathlessness and profuse diaphoresis within the last 2 hours. Physical examination revealed increased blood pressure and low oxygen saturation. Auscultation of the chest revealed bilateral basal pulmonary end‐inspiratory rales.

**Figure 1:**
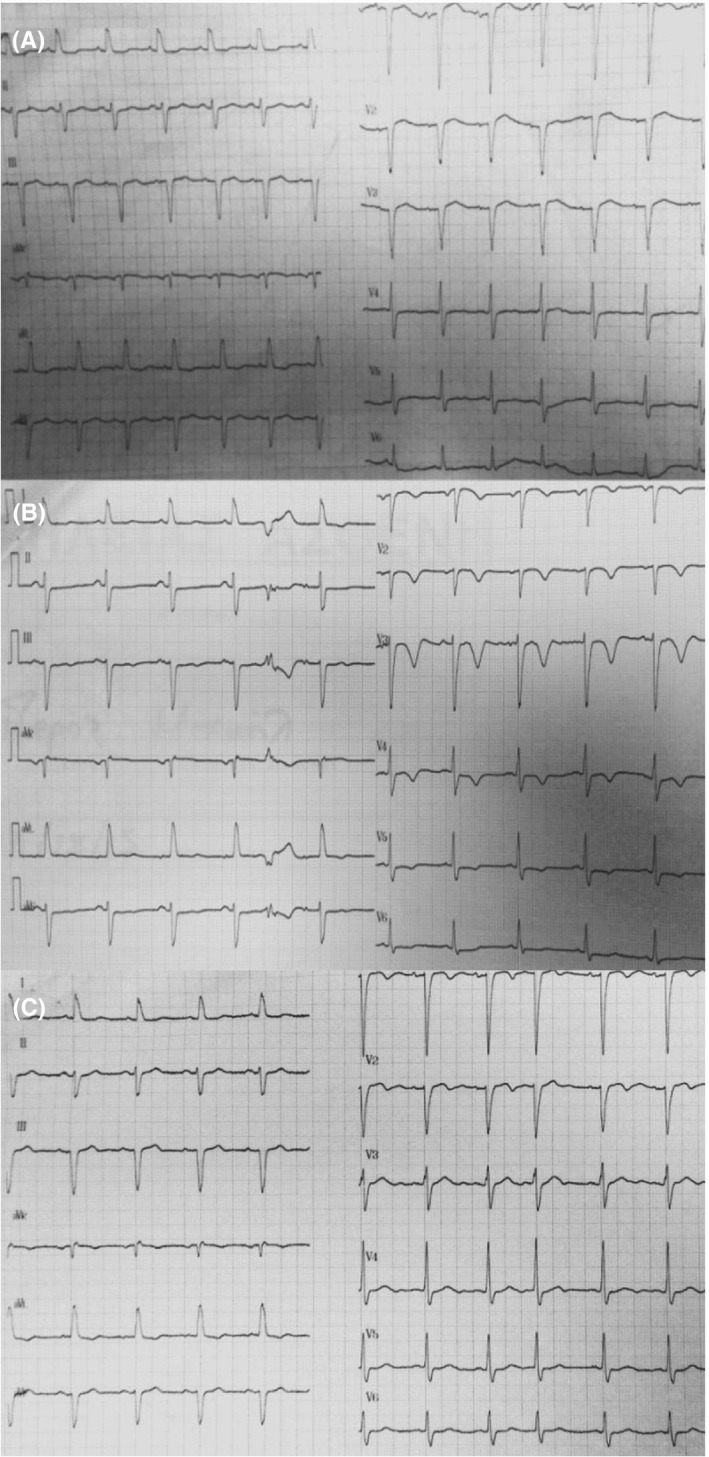
A, The 12‐lead surface electrocardiography (ECG) obtained on the admission did not demonstrate an acute myocardial injury pattern (small R‐waves in leads *V*_1_‐*V*_3_, without any ST‐segment elevation). B, On the second day of hospitalization, the 12‐lead surface ECG showed a diffuse T‐wave inversion in all precordial leads. C, On the fifth day of hospitalization, the ECG illustrates the complete resolution of T‐wave inversion in leads *V*_3_‐*V*_6_ and a partial resolution in leads *V*_1_‐*V*_2_

The electrocardiography (ECG) did not demonstrate an acute myocardial injury pattern (Figure 1A). Chest radiography showed bilateral diffuse infiltrations, Kerley B lines, and flow inversion. Cardiac serum markers (Architect STAT assay, high‐sensitivity troponin I; Abbott Diagnostics, Chicago, IL, USA) and D‐dimer test were negative. Echocardiographic assessment disclosed mild concentric hypertrophy, mildly impaired left ventricular systolic function without regional wall motion abnormalities and moderate diastolic dysfunction with elevated filling pressure.

A diagnosis of acute pulmonary edema was established, and the patient was hospitalized under closed monitoring in the intensive care unit. The acute heart failure symptoms were successfully subsided after administration of furosemide and glyceryl trinitrate intravenously, as well as angiotensin‐converting‐enzyme inhibitor orally in combination with oxygen support. Complete respiratory recovery occurred in approximately 12 hours.

**Figure 2:**
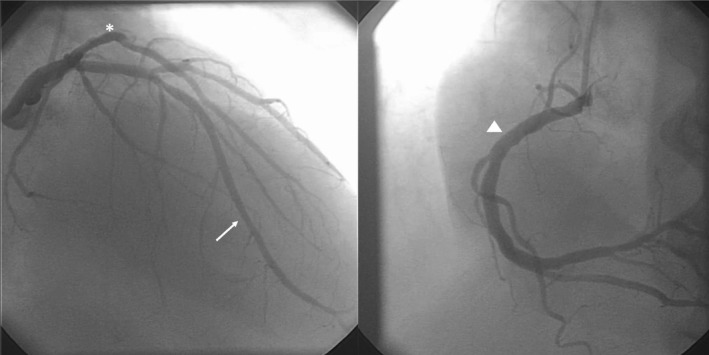
Coronary angiography revealed no significant coronary artery disease. Left anterior descending artery (arrow). Left circumflex artery (asterisk). Right coronary artery (triangle)

During the second day of hospitalization, the ECG showed a diffuse T‐wave inversion in all precordial leads (Figure 1B). The patient underwent coronary angiography, and significant coronary artery disease was ruled out (Figure 2). ECG T‐wave inversion gradually resolved within one week (Figure 1C).

To the best of our knowledge, only a few cases of late large T‐wave inversion after the occurrence of non‐ischemic pulmonary edema have been described.^1^, ^2^ Apart from myocardial ischemia, several well‐described causes may be associated with T‐wave inversion, including subarachnoid hemorrhage and hemorrhagic stroke, massive pulmonary embolism, pheochromocytoma, cocaine abuse, status epilepticus, gastrointestinal emergencies (perforated ulcer, acute pancreatitis, and acute cholecystitis), cardiac sarcoidosis, electroconvulsive therapy, and cardiac memory T‐wave pattern.^1^, ^2^ All the events above were excluded with regard to the patient's history and clinical manifestation.

## CONFLICT OF INTEREST

None declared.

## AUTHOR CONTRIBUTION

KT, NM: conception and design of the research and writing of the manuscript. CK, ES, MS: acquisition of data. ES, MS, CK, ER: analysis and interpretation of the data. HA, IP: critical revision of the manuscript for intellectual content.
